# Short-term acceptability of the Woman’s Condom and influencing factors among internal migrants in China

**DOI:** 10.1186/s12889-019-7625-0

**Published:** 2019-10-29

**Authors:** Junqing Wu, Junguo Zhang, Yuyan Li, Jinming Yang, Ying Zhou, Yiran Li, Shuangfei Xu, Rui Zhao

**Affiliations:** 1 NHC Key Lab. of Reproduction Regulation (Shanghai Institute of Planned Parenthood Research), Fudan University, Room 307, 779 old Humin Road, Xuhui District, Shanghai, 200032 People’s Republic of China; 2 Center for Clinical Epidemiology and Methodology (CCEM), Guangdong Second Provincial Central Hospital, Guangzhou, Guangdong 510000 People’s Republic of China; 3 NHC Key Lab. of Reproduction Regulation (Shanghai Institute of Planned Parenthood Research), Medical School, Fudan University, Shanghai, 200032 People’s Republic of China; 4 Department of Pharmacology, Vanderbilt University School of Medicine, 432 Preston Research Building, 23rd Avenue South at Pierce, Nashville, TN 37232-6600 USA; 5 School of Public Health, Fudan University, Shanghai, 200032 People’s Republic of China

**Keywords:** Woman’s Condom, Influencing factors, Internal migrants, Longitudinal study, Three-level model analysis

## Abstract

**Background:**

The Woman’s Condom, a newly designed condom for women, has obtained market approval in China, but it remains relatively unfamiliar to the migrant population. The aim of this study is to evaluate the short-term acceptability of the Woman’s Condom and influencing factors among internal migrants.

**Methods:**

A longitudinal study was conducted among 1800 migrants in Beijing, Chongqing, and Shanghai in China between August 2013 and August 2014.Three-level model was implemented with the Statistical Analysis System software (v.9.4 SAS Institute) to analyze within-individual changes, between-individuals effects, and between-group effects.

**Results:**

Three-level model analysis revealed statistically higher short-term acceptability of the Woman’s Condom among subjects who lived in Shanghai (*β* = 6.50, *t* = 2.76, *p* < 0.01), unmarried and not in a cohabiting relationship (*β* = 3.05, *t* = 2.76, *p* < 0.01) than those who lived in Beijing, married and in a cohabiting relationship. Female (*β* = − 1.69, t = − 7.55, *p* < 0.01) and lower educational attainment (*β* = − 2.30, t = − 1.94, *p* = 0.05) were negatively related, while occupations of education, health, and civil staff (*β* = 1.50, t = 2.92, *p* < 0.01) were positively related to acceptability. It was predicted that migrants’ acceptability of the Woman’s Condom would significantly increase over time (*β* = 1.09, t = 5.54, *p* < 0.01).

**Conclusions:**

The findings indicate that the Woman’s Condom enjoyed relatively high short-term acceptability among migrants in China. In order to popularize the Women’s Condoms in migrants, more publicity, consultation and training in open and prosperous areas should be strengthened.

## Background

Internal migrants in China, also called floating population, are residing in a location that is different from their place of household registration, which represents unique urban-rural segregation in China [1]. Since 1979, economic reform and rapid urbanization of China has resulted in significant rural-urban migration, which also has created abundant employment opportunities at the same time [2, 3]. By the end of 2015, China had the world’s largest mobile population, 247 million rural-to-urban migrants (18% of the total population) [4]. Most of the migrants were young and middle-aged adults, who were likely to be more sexually active than the general population [2, 5]. After leaving their homes, the migrants encountered more social and economic pressures and tended to be become isolated and anxious [2]. These changes may have caused risky sexual activities such as having unprotected sex, having multiple casual sexual partners, engaging in sex with commercial sex workers, and alcohol abuse [6]. As a result, migrants have been considered susceptible to HIV/AIDS and sexually transmitted infections (STIs) [7]. Thus, it is vitally important that measures should be taken to protect migrants from risky sexual activities.

The condom as a barrier method can effectively protect migrants against HIV/AIDS and STIs. In general, male condoms are highly acceptable and a good choice when men are willing to use them [8]. If not, gender inequality makes it difficult for women to have control over their partners’ decisions [8]. The female condom, an available female-initiated contraceptive method, is developed for use as the new condom option, especially when male condom cannot be used [9]. It has the potential to both empower women and please men [10]. Recent researches have shown that the female condom with trusted efficacy could increase the uptake of condom use, reduce stress caused by unintended pregnancy or STIs, and increase sexual pleasure and health benefits [11, 12]. Although female condoms have been marketed internationally since the mid-1990s, they remain relatively unpopular in China, for example, most migrants have never seen a female condom [13]. Also, limited geographical coverage, poor availability, high cost, and unattractive appearance have had a large impact on the introduction activities of the female condom in China [10, 13, 14]. More in-depth research is necessary on demographic, socioeconomic, and epidemiological factors, which will help to promote the use of the female condom in China and to introduce a new-generation female condom [13].

The Woman’s Condom, a new female-initiated protective product, has been developed by PATH, an international health organization, featuring an improved design [15]. It is designed through a user-centered development process to protect women and couples from unintended pregnancy, HIV/AIDS, and STIs, and to be highly pleasurable and more acceptable than the previous female products in China [16, 17]. A randomized crossover study in the USA involving 75 couples comparing the Woman’s Condom to the first-generation Female Condom showed that the Woman’s Condom had less failure, fewer adverse events, and higher acceptability [18]. Further, results from a randomized crossover trial among 170 women in Durban, South Africa, assessing the Woman’s Condom against the second-generation Female Condom, indicated that the Woman’s Condom was preferred over the second-generation Female Condom in appearance, ease of use and overall fit [19].

In 2010, the Woman’s Condom already obtained market approval in China [13]. Recently, several studies have explored the acceptability of the Woman’s Condom in China. A single-arm couples’ use study involving 60 couples was conducted in China in 2010. Huang et al. reported the Woman’s Condom had performed well in demonstrations in terms of condom function and safety [17], and Wu et al. assessed acceptability of the Woman’s Condom among the above population, confirming that the Woman’s Condom appeared to be acceptable [16]. However, Coffey et al. evaluated initial reactions to the Woman’s Condom by potential user groups, claiming that migrant men were less interested in using the Woman’s Condom possibly because they did not perceive a need for a dual-protection product [13]. Therefore, contradictory results may involve factors influencing the acceptability of the Woman’s Condom among internal migrants in China. In addition, previous study showed most participants were first-time Woman’s Condom users, and evaluation of participants’ acceptability was based on speculation, showing different results from repeat users [13]. We conducted a longitudinal study in three Chinese cities (Beijing, Chongqing, and Shanghai) to assess the short-term acceptability of the Woman’s Condom over time and to determine what influencing factors were related to its acceptability among internal migrants. In the longitudinal study, within-individual changes (the first time VS the fourth time), between-individuals effects (man and woman in the group), and between-group effects (things that are the same for the group such as city but vary between-groups) were evaluated. We hypothesized that the short-term acceptability of the Woman’s Condom might be influenced by the practice time, product safety, and group and individual characteristics.

## Methods

### Sampling strategy and study population

The longitudinal study about the short-term acceptability of the Woman’s Condom was conducted in three cities in China, namely Beijing, Chongqing, and Shanghai. Participants were recruited between August 2013 and August 2014. This research was a multi-stage sample designed to represent the internal migrants between 20 and 49 years of age in large Chinese cities.

Firstly, we selected three large cities including Beijing in north China, Chongqing in south-west China and Shanghai in south-east China, which represent different regions of China, and are the places where a great number of internal migrants flow into. Then, with the assistance of the family planning officials, we randomly selected two districts densely populated by migrants in each city.

Secondly, three types of locations including factory sites, construction sites, and service sites (including but not limited to, government organs, hospitals and malls), representing the places where the largest migrant population work, were surveyed in the selected districts. Several meetings and discussions were held with local governments and the family planning officials to compile lists including three types of locations in each district. According to the lists, quota-sampling was used to select investigation site in each district. Finally, four factory sites, four construction sites, and four service sites in each district of each city were selected.

Thirdly, awareness-raising educational sessions about sexual and reproductive health were held as the primary form of recruitment at the three types of locations including factories, construction sites, and service sites in the selected districts. At the end of the sessions, the study of the Woman’s Condoms was mentioned, and a brief description was given to the attendants. Interested migrants were invited to talk to the research team regarding participation and further confirmation whether to take part in this study. To be eligible for participation, subjects (1) were rural-to-urban migrants who had separate registered and actual residences, (2) aged 20–49 years, (3) had been living in the selected city for at least 6 months, (4) were healthy and sexually active, (5) were in a monogamous heterosexual relationship, (6) were not allergic to polyurethane or vaginal lubricants, (7) stated that their partners had also agreed and were willing to participate, and (8) were able and willing to participate in this investigation. In addition, female subjects could not be pregnant, seeking pregnancy, or breastfeeding. Finally, 600 eligible migrants (300 females and their partners) were enrolled to participate in each city.

### Study procedure

At the screening visit, a detailed explanation of the purpose and nature of this study was offered to eligible subjects who were willing to participate before the informed consent was obtained. Inquiries and physical examinations about reproductive health for subjects were performed to ensure that they had good general and genital health. Pregnancy tests for female migrants were done to ensure they were not pregnant. On a second visit, all the selected subjects in the same city were gathered in a private room at the factory or workplace to complete a self-administered demographic questionnaire. After ensuring the participation of eligible migrants, we conducted a training meeting for the subjects on the principle, effectiveness, possible side effects and complications, the use method and countermeasures when problems arise, and the role of prevention of unwanted pregnancy, RTI/STDs of Woman’s Condom. After finishing their training, each group (a woman and her partner) received four Woman’s Condoms, study questionnaires, and user instructions. The Woman’s Condoms were used at home four times over a month (once a week). Participants recorded any adverse effects after they used each condom, and returned their feedback to the researchers, who were able to provide proper counseling to them. After the first and fourth condoms were used, all subjects completed an acceptability questionnaire to rate their opinion about the Woman’s Condom. At the final visit, typically 4 weeks later, researchers collected the study questionnaires and conducted an exit interview with each migrant.

### Study device

The Woman’s Condom consists of an outer ring, a condom pouch, an insertion capsule, and four foam shapes. The condom pouch is made from polyurethane film with a thickness of only 0.03 mm. Four foam shapes, produced from hydrophilic urethane foam on the outside of the pouch, cling lightly to the vaginal wall to stabilize the device. The flexible, soft outer ring covers the external genitalia during sexual intercourse. The insertion capsule made of polyvinyl alcohol can dissolve once placed in the vagina. The Woman’s Condom is not pre-lubricated. A water-based and nonspermicidal lubricant should be used with the Woman’s Condom before intercourse.

### Assessment instruments

#### The short-term acceptability of the Woman’s Condom scale

In this study, a 19-item questionnaire was used to evaluate the short-term acceptability of the Woman’s Condom among migrants. On the basis of the theory of design and pre-investigation, using confirmatory factor analysis, the acceptability of the Woman’s Condom consists of three aspects: ease of use, comfort, and satisfaction. Three factors respectively contained 6, 7, and 6 items with response categories ranging from 1 = very difficult/uncomfortable/unsatisfied to 5 = very easy/comfortable/satisfied. This scale had been well designed to assess the acceptability among 60 married couples in Shanghai [16]. The value of Cronbach’s alpha for this scale was 0.98 in the current sample. Finally, the sum score with a range between 0 and 95 of the acceptability scale based on the participants’ subjective opinions was regarded as the outcome of the study.

#### Potential influencing factors

In this study, a woman and her partner were regarded as one group. A longitudinal structure, also called a three-level structure, involved 900 groups, two individuals in each group, and two time points in each individual. Then characteristics of group level, between-individual level, and within-individual level of selected migrant subjects were collected as potential factors in the acceptability of the Woman’s Condom. Variables analyzed are shown as follows.

Between-groups characteristics included investigation areas (Beijing, Chongqing, Shanghai), number of children (0, 1, > = 2), time being together (less than one year, one–three years, longer than three years), marital status (married and live together, married but do not live together, unmarried and do not live together, unmarried but live together), and contraception in the last month (no use, intrauterine device, oral contraceptive, male condom, and others).

Between-individual characteristics were measured in terms of age (years, continuous), gender (male/female, dichotomous), ethnicity (Han Chinese/others, dichotomous), educational attainment (junior school or below, high school, three-year college, bachelor’s degree or above), occupation (unemployed or self-employed, workers/salespeople, education, health and civil servants, others), and adverse effects from the Woman’s Condom (yes/no, dichotomous). Adverse effects from the Woman’s Condom included genital itching, burning, stinging, pressure while urinating, genital pain/discomfort, and genital irritation/rash, which were recorded by participants after each condom use. Because of a low adverse rate, four-time records were summarized as a binary variable, which was defined by whether subjects had suffered adverse effects from the Woman’s Condom in this study or not.

A within-individual effect was assessed by practice time, which included two time points (the first time and the fourth time).

### Data analysis

Descriptive statistics on the selected migrant population used in this research were as the mean and standard deviation (SD) for continuous variables, while frequencies and percentages were presented for categorical variables. The mean scores and SD of the first use and the fourth use, and their changes, were calculated at each level of categorical variables. The score differences were tested by t-tests or analyses of variance (ANOVA), or Wilcoxon tests or Kruskal-Wallis when appropriate.

We constructed a three-level model to examine growth in mean score of acceptability over time. Firstly, we examined the strength of the intra-class correlation coefficient using the null model. Additionally, we explained between-group variability using a level-3 covariate, and between-individual variability using a level-2 covariate, and further added a level-1 covariate. Moreover, random slopes for each level covariate were checked. In all models, after adjusting for covariates and the variance-covariance structure, random effects were not significant. At last, based on prior knowledge and significance of variables, a well-designed and simplified model was determined and used to explain the aim of this study. These statistical analyses were implemented with the Statistical Analysis System software (v.9.4 SAS Institute, Cary, NC, USA). The significance level at *P* < 0.05 was set, and all test hypotheses were two-sided.

## Results

The information of potential factors with the Woman’s Condom is summarized in Table 1. Of these 1800 participants, 97.7% were Han Chinese. The average age of participants was 34.79 (SD = 6.59) years old. For education level, 48.0% of the participants had obtained a bachelor’s degree or higher, and 30.9% had graduated from a three-year college. Participants were employed as workers/salespeople (21.0%); education, health, and civil staff (50.8%); or in other occupations (10.8%). The majority of women (93.3%) and their partners had married and lived together. Most of them (71.9%) had maintained their current relationship for longer than 3 years, and about 72.2% of them had one child. The current contraceptive method used by most participants was the male condom (54.0%), followed by oral contraceptives (24.7%), and 2.0% of participants reported no recent contraceptive use. Only 1.8% of adverse effects, mainly genital itching, were reported during the study.

**Table 1:** The descriptive statistics of potential factors with the Woman’s Condom among selected migrants

Between-individuals effects	*N* (%)
Age (years)	34.79 ± 6.59
Gender	
Male	900 (50.00)
Female	900 (50.00)
Ethnicity	
Han Chinese	1754 (97.66)
Others	42 (2.34)
Education	
Junior or below	94 (5.25)
High school	284 (15.85)
Three-year college	554 (30.91)
Bachelor degree or above	860 (47.99)
Occupation	
Unemployed or self-employed	312 (17.33)
Workers/salespeople	378 (21.01)
Education, health and civil staff	915 (50.83)
Others	195 (10.83)
Between-groups effects	N (%)
Residence	
Beijing	600 (33.33)
Chongqing	600 (33.33)
Shanghai	600 (33.34)
Number of children	
0	396 (22.32)
1	1280 (72.15)
≥ 2	98 (5.52)
Time being together	
Less than 1 year	188 (10.48)
1–3 years	316 (17.61)
Longer than 3 year	1290 (71.91)
Marital status	
Married and live together	1672 (93.30)
Married but do not live together	38 (2.12)
Unmarried and do not live together	38 (2.12)
Unmarried but live together	44 (2.46)
Contraception in the last month	
No use	36 (2.00)
Intrauterine device	258 (14.33)
Oral contraceptive	444 (24.67)
Male Condom	972 (54.00)
Others	90 (5.00)

*Abbreviations*: *N (%)* Number of subjects/Percentages, *SD* Standard deviation

### Univariate analyses of short-term acceptability at the first use and the fourth use and the change

As shown in Table 2, the mean score of short-term acceptability of the first use and the fourth use and the change were 57.31 (SD = 14.19), 58.35 (SD = 15.22), and 1.09 (SD = 8.18), respectively. The fourth-use mean score was higher than the first-use mean at each level of influencing factors except the level of Chongqing subjects. In the first use, the acceptability of the Woman’s Condom differed significantly in place of residences and marital status (*p* < 0.05). Different jobs seem to be associated with rates of acceptability of the fourth use that are different from those unemployed or self-employed (*p* < 0.05). Furthermore, migrants who had higher first-use and fourth-use acceptability scores were more likely to be male, who lived in Shanghai or Beijing rather than Chongqing, and who had recently used contraceptive methods. Results also revealed that the change of the mean score between first use and fourth use differed in terms of age, occupation, and place of residences (*p* < 0.05).

**Table 2:** Comparation of mean scores and changes of the acceptance of the Woman’s Condom by demographic characteristics

Variables		*N* (%)	The acceptability of the Woman’s Condom					
			First-use mean (SD)	*p* value*	Fourth-use mean (SD)	*p* value*	Change^a^	*p* value*
Between-individuals effects								
Age (Mean ± Standard Deviation)		34.79 ± 6.59	57.31 ± 14.19	0.79	58.35 ± 15.22	0.26	1.09 ± 8.18	0.04
Gender	Male	900 (50.00)	58.06 ± 13.97	0.02	59.30 ± 15.05	0.01	1.25 ± 8.40	0.37
	Female	900 (50.00)	56.57 ± 14.38		57.41 ± 15.34		0.94 ± 7.96	
Ethnicity	Han Chinese	1754 (97.66)	57.35 ± 14.21	0.35	58.39 ± 15.24	0.44	1.07 ± 8.20	0.60
	Others	42 (2.34)	55.63 ± 13.32		56.76 ± 14.42		2.03 ± 7.47	
Education	Junior or below	94 (5.25)	58.53 ± 13.21	0.26	58.35 ± 15.89	0.25	0.35 ± 10.18	0.71
	High school	284 (15.85)	57.78 ± 15.47		58.93 ± 16.09		1.37 ± 7.80	
	Three-year college	554 (30.91)	56.77 ± 13.68		57.50 ± 15.24		0.76 ± 8.24	
	Bachelor degree or above	860 (47.99)	57.37 ± 14.23		58.71 ± 14.88		1.31 ± 8.03	
Occupation	Unemployed or self-employed	312 (17.33)	56.39 ± 13.74	0.83	56.28 ± 15.27	0.01	0.01 ± 10.52	0.01
	Workers/salespeople	378 (21.01)	58.20 ± 15.33		59.25 ± 16.67		1.09 ± 7.65	
	Education, health and civil staff	915 (50.83)	57.48 ± 13.96		58.55 ± 14.64		1.03 ± 7.43	
	Others	195 (10.83)	56.19 ± 13.58		58.94 ± 14.70		3.15 ± 7.97	
Adverse effects	Yes	32 (1.78)	57.49 ± 14.11	0.01	58.47 ± 15.13	0.11	1.06 ± 8.15	0.17
	No	1768 (98.22)	46.83 ± 15.07		50.50 ± 18.96		3.35 ± 9.85	
Between-groups effects								
Residence	Beijing	600 (33.33)	57.03 ± 13.87	0.01	59.39 ± 13.96	0.01	2.66 ± 7.92	0.01
	Chongqing	600 (33.33)	54.33 ± 13.74		53.36 ± 15.34		−0.92 ± 9.12	
	Shanghai	600 (33.34)	60.62 ± 14.27		62.39 ± 14.91		1.65 ± 6.88	
Number of children	0	396 (22.32)	58.56 ± 15.54	0.36	59.58 ± 15.99	0.83	1.10 ± 8.88	0.54
	1	1280 (72.15)	57.10 ± 13.97		58.08 ± 15.10		0.99 ± 7.88	
	> = 2	98 (5.52)	55.24 ± 11.20		57.29 ± 13.58		2.53 ± 9.12	
Time being together	Less than 1 year	188 (10.48)	59.83 ± 15.11	0.07	59.96 ± 16.29	0.44	0.24 ± 10.55	0.73
	1–3 years	316 (17.61)	57.83 ± 15.80		59.03 ± 16.63		1.18 ± 8.21	
	Longer than 3 year	1290 (71.91)	56.79 ± 13.59		57.93 ± 14.67		1.20 ± 7.77	
Marital status	Married and live together	1672 (93.30)	57.30 ± 14.11	0.01	58.37 ± 15.07	0.06	1.13 ± 8.18	0.28
	Married but do not live together	38 (2.12)	58.82 ± 14.12		59.32 ± 18.56		0.14 ± 10.47	
	Unmarried and do not live together	38 (2.12)	61.11 ± 16.50		61.77 ± 16.13		0.66 ± 6.84	
	Unmarried but live together	44 (2.46)	50.35 ± 12.41		51.34 ± 15.41		0.78 ± 7.84	
Contraception in the last month	No use	36 (2.00)	49.64 ± 19.92	0.01	51.69 ± 21.52	0.01	1.97 ± 10.95	0.11
	Intrauterine device	258 (14.33)	57.55 ± 14.33		59.16 ± 15.81		1.62 ± 6.77	
	Oral contraceptive	444 (24.67)	59.95 ± 12.71		61.33 ± 13.58		1.31 ± 7.07	
	Male Condom	972 (54.00)	56.42 ± 14.23		56.95 ± 15.35		0.66 ± 8.95	
	Others	90 (5.00)	56.07 ± 15.72		59.08 ± 14.19		2.91 ± 6.58	

*Abbreviations*: *N (%)* Number of subjects/Percentages, *SD* Standard deviation

*The *P* values were by t-test or analysis of variance (ANOVA) or Wilcoxon test or Kruskal-Wallis where appropriate

^a^The changes between the first-use and the fourth-use were calculated by the scores of fourth-use minus the scores of first-use

### Variability in short-term acceptability over time using a three-level model analysis

Multilevel analyses examined the variability and relationship between short-term acceptability and influencing factors on the between-group (level 3), between-individual (level 2), and within-individual levels (level 1) (Table 3). Random effects showed significant between-group variance for the intercept (z = 19.87, *p* < 0.01), between-individual variation of the intercept (z = 4.92, *p* < 0.01), and significant within-subject variability (z = 33.18, *p* < 0.01).

**Table 3:** Association between potential factors and the acceptability of the Woman’s Condom

Fixed effects	Estimate (SE)	*t*	*p* value
Intercept	56.43 (2.03)	27.74	< 0.01
Between-groups effects			
Residence			
Beijing	Reference		
Chongqing	−4.99 (1.13)	−4.42	< 0.01
Shanghai	3.05 (1.11)	2.76	< 0.01
Marital status			
Married and live together	Reference		
Married but do not live together	0.98 (3.10)	0.32	0.75
Unmarried and do not live together	6.50 (2.91)	2.23	0.03
Unmarried but live together	−5.37 (3.10)	−1.73	0.08
Between-individuals effects			
Gender			
Male	Reference		
Female	−1.69 (0.22)	−7.55	< 0.01
Educational attainment			
Junior or below	Reference		
High school	−1.43 (1.10)	−1.30	0.19
Three-year college	−1.62 (1.15)	−1.41	0.16
Bachelor degree or above	−2.30 (1.19)	−1.94	0.05
Occupation			
Unemployed or self-employed	Reference		
Workers/salespeople	0.91 (0.56)	1.64	0.10
Education, health and civil staff	1.50 (0.51)	2.92	< 0.01
Others	0.51 (0.73)	0.70	0.48
Adverse effects from the Woman’s Condom			
Yes	Reference		
No	3.07 (1.45)	2.11	0.04
Within-individuals effect			
Practice time			
The first time	Reference		
The fourth time	1.09 (0.20)	5.54	< 0.01
Random effects	Estimate (SE)	*z*	*p* value
Level 3 (VAR (*int*))	168.13 (8.46)	19.87	< 0.01
Level 2 (SP (EXP))	0.33 (0.07)	4.92	< 0.01
Level 1 (Residual)	35.35 (1.07)	33.18	< 0.01

*Abbreviations*: *SE* Standard Error

Results revealed significant associations between acceptability and between-group differences in residences and marital status, indicating that women and their partners in Shanghai (*β* = 3.05, t = 2.76, *p* < 0.01) who were not married and not living together (*β* = 6.50, t = 2.76, *p* < 0.01) reported higher acceptability scores than groups in Beijing who were married and lived together. However, groups in Chongqing (*β* = − 4.99, t = − 4.42, *p* < 0.01) had lower acceptability scores than groups in Beijing.

In addition, between-individual differences in gender, educational attainment, occupation, and adverse effects caused by the Woman’s Condom were significantly associated with acceptability. Women tended to have lower acceptability scores than men (*β* = − 1.69, t = − 7.55, *p* < 0.01). Subjects who were employed as education, health, and civil staff had higher acceptability scores than freelancers (*β* = 1.50, t = 2.92, *p* < 0.01), but subjects who obtained bachelor’s degrees or above had lower acceptability scores than subjects who were only junior middle school graduates or below (*β* = − 2.30, t = − 1.94, *p* = 0.05). Migrants who had not suffered adverse effects from the Woman’s Condom could increase their acceptability scores (*β* = 3.07, t = 2.11, *p* < 0.01).

Within-individual variability in time was identified as a significant predictor for acceptability. Results indicated that the mean score of acceptability significantly increased over time (*β* = 1.09, t = 5.54, *p* < 0.01).

## Discussion

Since the female condom was introduced, many studies have found that increasing use of the female condom could decrease the number of unprotected sexual encounters, pregnancy rates, and STIs [20, 21]. Research on the acceptability of the female condom and its influencing factors have been documented among sex workers, couples, patients from clinics, and volunteers. However, these studies neither have used the newly designed premium Woman’s Condom nor have focused on internal migrants in China. Furthermore, because of variable factors and cultural backgrounds, approaches used in those studies influenced the acceptability of the condom, and led to inconsistent results and obscured the conclusion [20]. In the current study using three-level model analysis of the short-term acceptability of the Woman’s Condom scale, we quantitatively evaluated acceptability among migrants and further explored the relationship between the acceptability and related influencing factors among the migrant population in three cities of China. We observed that gender, occupation, educational attainment, marital status, residence, adverse effects, and use frequency were significantly associated with acceptability among women. This study is the first to use a longitudinal method based on multilevel models to analyze the Woman’s Condom’s acceptability among migrants and potential influencing factors.

Recently, a few studies reported their investigation in the acceptability of the Woman’s Condom in China since the female condom has been a less popular product in the market. One study reported that the Woman’s Condom was perceived as more pleasurable than male condoms [13]. Another study indicated that compared to the male condoms, the Woman’s Condom was two-fold preferred by American males, and 2.6-fold preferred by American females [18]. Moreover, the Woman’s Condom was generally acceptable among South Africans because of its better appearance, feeling, ease of use, and overall fitness [19]. In the current study, the mean first- and fourth-use acceptability scores of the Woman’s Condom were 57.31 and 58.35 respectively. Compared to local married couples, migrants show similar acceptability of the Woman’s Condom for the first use but have a lower score for the fourth use [16]. Based on the empirical results, our research indicated that migrants in China might show relatively high acceptability of the Woman’s Condom but had a lower rate of increase in acceptability than local residents over time.

In many studies, gender was found to be significantly associated with acceptability. Our finding was in agreement with the theory, and we further found that males would be more willing to use condoms than females. In some countries, male partners’ objection was the most commonly cited factor preventing initial and continued use [22, 23]. However, this study revealed that males expressed more openness toward this new type of condom than females. In China, women participants were more skeptical about using the Woman’s Condom than male participants, and they expressed less interest in the Woman’s Condom than in other contraceptive methods [13]. Since women have obtained more potential control over their lives and relationships, the use of the Woman’s Condom might depend largely on women’s willingness [24]. Thus, to promote acceptability in migrants, both men and women’s interest and willingness should be motivated.

In addition, some studies have revealed a correlation between acceptability and age, but with confused results [13]. In terms of age, older females in the USA and South Africa were more likely to accept and use the female condom than younger women [25, 26]. However, an interesting study found that younger women were likelier than older women to prefer the female condom in Zimbabwe [27]. In the current study, we observed no obvious association between age and acceptability, but there was a significant correlation between age and mean score based on the analysis for the initial users and fourth-time users. The acceptability of the Woman’s Condom increased along with the age, possibly because aging women might have more knowledge of reproductive anatomy and be more accepting of touching their own genitals, which might help them to practice female condom insertion and removal more effectively [28].

Socioeconomic status was measured using occupation and educational attainment. Migrants who were employed as education, health, and civil staff, or had lower education levels were more likely to accept the Woman’s Condom. Zaman et al. reported that subjects who stated that they were pharmacists or doctors were more likely to accept the female condom [24]. Compared with those unemployed or self-employed, well-paid jobs could empower subjects to have the right to decide their lives. One of the US studies also found that low levels of educational attainment were associated with high acceptability and use [20]. This may be a reflection of the greater ability of more educated people to negotiate male condom use and not to necessitate a female-controlled method [25, 29].

Much researches have indicated that marital status was correlated with acceptability of the female condom. In Zimbabwe and the US, subjects agreed that female condoms would be an acceptable contraceptive method within marriage and other stable relationships [10, 30]. It might be easier for a person to introduce and try the female condom with a regular partner [20]. Together with other studies, our result is in agreement with these findings and further demonstrates that migrants who were unmarried and not living together with their partners would be more likely to use the Woman’s Condom than married couples. It is possible that single migrants were younger and engaged in more frequent sex so that they had greater concern about avoiding diseases [31]. The investigation site as an important contextual factor often had a greater impact on acceptability and use [22, 25]. This study also found higher acceptability of the Woman’s Condom among migrants living in Beijing or Shanghai than among floating populations in Chongqing. Until 2015, 10.3, 14.0 and 5.6% internal migrants resided in north China, south-east China, and south-west China, of which representative cities are Beijing, Shanghai and Chongqing, respectively. Compared to Beijing and Shanghai, Chongqing located in inland has not much economic prosperity and opening-up social atmosphere. The results presented above hypothesized that the higher prevalence of STIs, and a more open and prosperous environment would promote migrants’ acceptability of a new contraceptive method to protect themselves.

Adverse effects caused by female condoms would decrease the acceptability of the Woman’s Condom, but incidence of such effects was generally very low [12]. Several studies reported a range of adverse effects from < 2.0% to < 4.0% for various types of female condoms [12, 32]. In the current study, only 1.8% of adverse effects from the Woman’s Condom were reported. This result was not only depended on the proper design of the Woman’s Condom, but also relied on proper training and practice during the study. This study further noted that the acceptability of the Woman’s Condom was increasing over time. The underlying theory for the result is that “practice makes perfect,” which means that overall acceptability improves over time with instruction and skills training, regardless of condom type [18, 33]. A number of studies confirmed this theory and indicated that most participants overcame initial difficulties with practice, resulting in high acceptability and few adverse effects [22, 33]. No evidence of the association between contraception in the last month and the acceptability was observed . It is possible that the initial difficulties of the Woman’s Condom did not make it less attractive than other methods of contraception.

Based on this study, we further recognized that, in order to popularize the Women’s condoms in migrants, we should select more open and prosperous areas as the first large-scale promotion site, expand the scope of publicity and offer consultation and training to migrants during extension phase, thereby to better improve the acceptability of the Women’s Condoms by the migrants.

A limitation of this study should be determined. Although the study has analyzed many factors potentially influencing acceptability of the Woman’s Condom, factors such as cost, availability and access were not evaluated because it was only in the recent years that the Woman’s Condom has entered the Chinese market. Further research should include these factors to assess acceptability.

## Conclusion

Our research suggests that the Woman’s Condom enjoyed relatively high short-term acceptability among migrants in China, and the short-term acceptability was affected by within-individuals effect (practice time), between-individuals effects (gender, occupation and adverse effects), and between-groups effects (place of residence and marital status).

## Data Availability

The datasets generated and analyzed for this study are not publicly available due to participant privacy but are available from the corresponding author upon reasonable request.

## Abbreviations

ANOVA: Analysis of variance
SD: Standard deviation
STIs: Sexually transmitted infections
